# The application of Matching Law in exploring obsessive-compulsive behaviour: A case-control study

**DOI:** 10.1192/j.eurpsy.2025.703

**Published:** 2025-08-26

**Authors:** A. Hassoulas, P. Reed, L. McHugh

**Affiliations:** 1 School of Medicine, Cardiff University, Cardiff; 2 Swansea University, Swansea, United Kingdom; 3 University College Dublin, Dublin, Ireland

## Abstract

**Introduction:**

The rigid maintenance of an acquired response in participants with obsessive-compulsive (OC) traits has been widely reported in the literature (e.g., Hassoulas et al., 2014; 2017; 2024). As such, demonstrating flexibility in responding to concurrently presenting stimuli with differing rates of reinforcement has also been shown to be difficult for those diagnosed with OCD (e.g., Clayton et al, 1999). Matching Law proposes a quantifiable description of behaviour when consequences to responding under two concurrently presented schedules of reinforcement differ. The application of a matching procedure, whereby participants exhibiting OC traits are presented with concurrent schedules of reinforcement, would highlight this difficulty in distinguishing between stimuli that provide different rates of reinforcement. That is, the competing sources of reinforcement would relate to the concurrent stimuli that individuals with OCD have been shown to have trouble attending to separately.

**Objectives:**

Sensitivity to concurrent schedules has not been previously investigated in the context of OCD. As such, two experiments were designed to measure matching behaviour between groups of participants with and without OC traits.

**Methods:**

A total of 60 participants (33 females, 27 male) were recruited to take part in the two studies. The Maudsley Obsessive-Compulsive Inventory (MOCI) was used to screen for the presence of OC traits. Concurrent variable intervals (VI) were presented over ten trials, with an aversive auditory stimulus serving as a punisher superimposed during every second trial. During the first experiment, VI 20-second schedules were concurrently presented with VI 40-second and VI 60-second schedules. The ratio between concurrent schedules was widened during the second experiment; with VI 20-second schedules presented alongside VI 100-second and VI 180-second schedules.

**Results:**

A significant interaction of trial with OC traits was revealed when the punisher was presented in experiment 1, *F*(4,68)=2.68, *p < 0.05,* but not in experiment 2 (p>0.05). The findings suggest that the presence of an auditory punisher facilitates schedule-sensitive responding in participants with OC traits, but that schedule-sensitivity is lost when intervals between concurrent schedules are widened. Figures 1 and 2 display response ratios for both high and low scoring groups during experiments 1 and 2 respectively.

**Image 1:**

**Figure:**
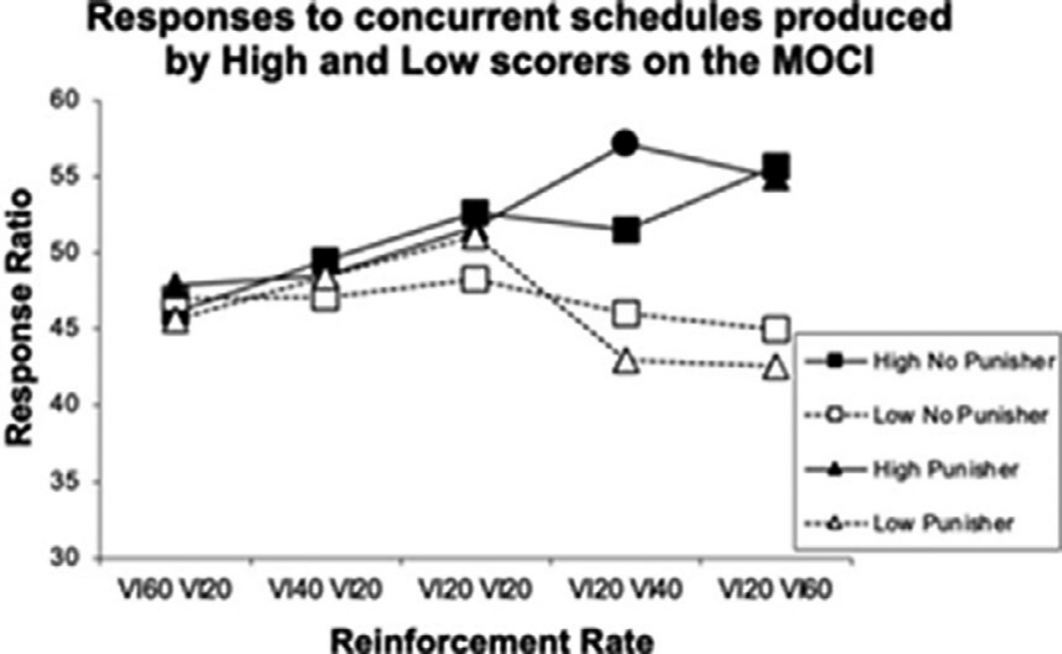

**Image 2:**

**Figure:**
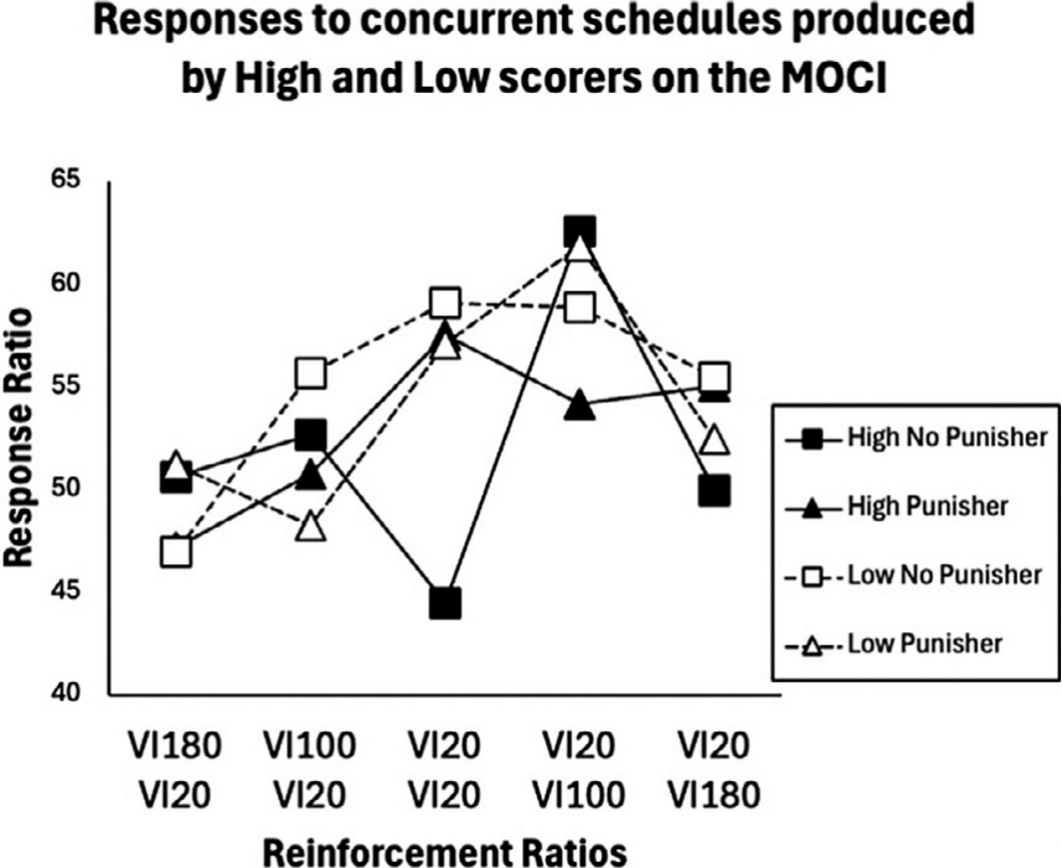

**Conclusions:**

The widening of the schedule ratios in the second experiment disrupted sensitivity to the schedule contingencies for the OC group. Greater flexibility in responding within this group can therefore be facilitated by considering specific parameters and rates of reinforcement.

**Disclosure of Interest:**

None Declared

